# Zebrafish Embryo Vessel Segmentation Using a Novel Dual ResUNet Model

**DOI:** 10.1155/2019/8214975

**Published:** 2019-02-03

**Authors:** Kun Zhang, Hongbin Zhang, Huiyu Zhou, Danny Crookes, Ling Li, Yeqin Shao, Dong Liu

**Affiliations:** ^1^School of Electrical Engineering, Nantong University, Nantong 226019, China; ^2^Department of Informatics, University of Leicester, Leicester, UK; ^3^ECIT, Queen's University, Belfast, UK; ^4^School of Computing, University of Kent, Canterbury, UK; ^5^School of Transportation, Nantong University, Nantong 226019, China; ^6^Co-innovation Center of Neuroregeneration, Jiangsu Key Laboratory of Neuroregeneration, Nantong University, Nantong 226001, China

## Abstract

Zebrafish embryo fluorescent vessel analysis, which aims to automatically investigate the pathogenesis of diseases, has attracted much attention in medical imaging. Zebrafish vessel segmentation is a fairly challenging task, which requires distinguishing foreground and background vessels from the 3D projection images. Recently, there has been a trend to introduce domain knowledge to deep learning algorithms for handling complex environment segmentation problems with accurate achievements. In this paper, a novel dual deep learning framework called Dual ResUNet is developed to conduct zebrafish embryo fluorescent vessel segmentation. To avoid the loss of spatial and identity information, the U-Net model is extended to a dual model with a new residual unit. To achieve stable and robust segmentation performance, our proposed approach merges domain knowledge with a novel contour term and shape constraint. We compare our method qualitatively and quantitatively with several standard segmentation models. Our experimental results show that the proposed method achieves better results than the state-of-art segmentation methods. By investigating the quality of the vessel segmentation, we come to the conclusion that our Dual ResUNet model can learn the characteristic features in those cases where fluorescent protein is deficient or blood vessels are overlapped and achieves robust performance in complicated environments.

## 1. Introduction

**Figure 1:**
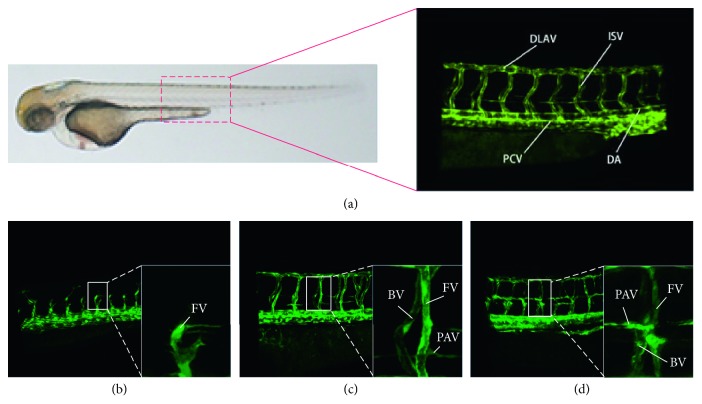
Zebrafish fluorescent vascular 2D view: (a) natural pattern and enlarged view of trunk (DLAV: dorsal; ISV: intersegmental vessel; PCV: posterior cardinal vein; DA: dorsal aorta); (b–d) the zebrafish embryo's trunk vessels of 35H, 48H, and 72H, respectively (FV: foreground vessel; BV: background vessel; PAV: parachordal vessel).

The genome of zebrafish is 87% homologous to the human genome, and its tumorigenesis process is very similar to human tumor [1]. Comparing the human genome sequence with the completed genome sequence of zebrafish, it is found that zebrafish has a high degree of consistency in oncogenes, tumor suppressor genes, and cell-cycle regulation genes [2]. Therefore, zebrafish is widely used as a model to study the pathogenesis of diseases such as tumors, cardiovascular diseases, and nervous system diseases, as well as the dynamic mechanisms of related drugs. Researchers have successfully constructed fusion genes containing green fluorescent protein (G-RCFP) genes [3, 4]. These genes can specifically express green fluorescent proteins and present blood vessels with green fluorescent proteins during the angiogenesis of zebrafish. With the help of confocal microscopy, the whole process of angiogenesis can be observed directly, and the angiogenesis of zebrafish before and after administration is carried out. Compared with other blood vessels, intersegmental vessel (ISV) angiogenesis in zebrafish embryos is more susceptible to drugs and genes and presents different morphological features (short, adhesional, thick, and branching), so it is an ideal model for studying drugs and disease pathology. High-throughput screening using human observation has presented issues such as low efficiency and inconsistent outcomes. If researchers can design an automated zebrafish vessel segmentation system, the efficiency of neural computation can be greatly improved, and the professionals can be freed up to handle more challenging scenarios. However, as far as we know, there is a lack of in-depth research on the segmentation of zebrafish fluorescence vessels. Zebrafish's natural patterns and the projection map of fluorescent vascular models are shown in Figure 1. The fluorescence blood vessels of zebrafish embryos are 3D models, obtained by using a laser confocal microscope.

In Figure 1, due to the lack of spatial information, a pair of ISVs appears to overlap in a dense region. Compared to the occluded background ISV, the foreground ISV retains complete shape information, and our task is to segment the foreground ISV region for subsequent processing. The abnormal shapes of ISV consist of missing structures, irregular branches, and thick pillars. Figure 1(b) shows a zebrafish vascular model that grew for 36 hours with immature development and short blood vessels. From Figure 1(c), we can see that the ISV has developed and formed a complete morphology, and although there is a small PAV, it does not affect the final outcome. Figure 1(d) shows the vascular model of the zebrafish growing for 72 hours. As a result of vascular overgrowth, it is difficult to distinguish from the branch adhesion in abnormal situations as there exists a lot of parachordal vessels, which interfere with the vascular model. Therefore, we can select the zebrafish embryo vascular model with a culture time of 48 hours. The separation of the foreground ISV from the complex environment is the key to investigating the whole zebrafish vascular model system. However, the state-of-the-art segmentation methods [5–8] cannot fully solve certain practical problems: (a) ISV has overlapping areas of the same fluorescence intensity and (b) ISV has uneven fluorescence intensities.

In this paper, we propose a dual U-Net bridged architecture. U-Net is a classic encoder-decoder network that has achieved exciting results in medical image segmentation applications [5]. In particular, we connect each decoder module of the first U-Net to the corresponding encoder module of the second U-Net so that the features of the previous layer can be linked with those of the later layer. Meanwhile, we introduce a shape-constraint to the contour-aware segmentation loss function in order to handle the difficulty of segmentation caused by various complex forms and uneven fluorescence intensities.

The main contributions of our work are as follows:

- The application of convolution neural networks in the segmentation of zebrafish angiofluorescence vessels is reported for the first time. Compared to other zebrafish vessel processing methods, we provide a new idea for automated identification of normal and abnormal vessel development, filling the gap between the low-level features and the high-level segmentation.
- To combine the advantages of the original U-Net and the full preactivation Residual Unit [9], we propose a novel segmentation model that achieves higher segmentation accuracy.
- We introduce a novel shape-constraint term into the contour-aware segmentation loss function for fine-grained segmentation in complex situations.

## 2. Related Work

### 2.1. Zebrafish Segmentation Methods

Ip et al. [10] proposed a method for segmenting the tail blood vessels of zebrafish embryos. This method effectively reconstructs blood vessels in various directions to distinguish different blood vessel types. Based on this study, Feng et al. [11] proposed a framework for extracting blood vessels and calculating vascular morphological parameters. Based on the prior knowledge of image sequences, their algorithm uses edge tracking and curve fitting to detect the contours of the tail and tail veins. Based on Haar-like features and the improved AdaBoost cascade classifier, Yang and Xu [12] reported the identification of internode blood vessels. However, there is no further analysis of interstitial blood vessels. Zhao et al. [13] proposed a method for automatically detecting defective zebrafish embryos, extracting the zebrafish outline into graphics, and using texture descriptors and morphological operations to segment the zebrafish from the background. This is a method of binary classification of embryo shapes. However, these methods do not involve the segmentation of fluorescent blood vessels in zebrafish embryos.

### 2.2. Deep Learning Methods

CNN is one of the most advanced technologies in image classification, and given the availability of a large training dataset, it can be used to study image characteristics. In our original data, the zebrafish vascular models are 3D vascular models. It is worth noting that the 3D convolution network architecture used for medical model segmentation is widely used [14–17]. However, in our ISV dataset, there are often discrete blood vessels or fluorescent proteins present in non-blood vessels, resulting in poor spatial and contextual information. Using 3D networks adds complexity, as the convolution filters and internal representations have additional dimensions.

In recent years, semantic segmentation is one of the key problems in computer vision. Long et al. [18] proposed a leaping architecture that combines semantic information from deep layers with characterization information from shallow layers to produce accurate and detailed segmentation. He adapted the classic classification network (AlexNet [19], the VGGNet [20], and GoogLeNet [21]) and passed their learning performance to the segmentation task by fine-tuning [22]. However, in the actual segmentation, because of the fixed receptive field, oversized targets may not be detected or may be discontinuous, small targets are easily ignored, and details of the object are often lost or smoothed. In order to solve these problems, Noh et al. [23] proposed a deep deconvolution network, based on VGG16. The network uses multiple proposals for each image to predict and then aggregates these to get the final prediction. Badrinarayanan et al. [24] proposed a deep convolutional coding and decoding architecture for image segmentation. This framework consists of a coding network and a corresponding decoding network, followed by a pixel-level classification layer. The architecture of the encoder network is similar to the convolutional layer of VGG16. The role of the decoding network is to map the low resolution-encoded feature map to the feature map of the input resolution. The FC layer is removed, resulting in a significant reduction in parameters and a much faster training speed.

Segmentation of medical images often takes shape information into account to produce reliable results, since otherwise, significant noise information will be captured. Analytical methods based on shape features have been used in medical image segmentation [25–27]. For example, Alvén et al. [28] proposed a method of shape perception label fusion, which is different from label fusion at the voxel level. Each atlas was considered as the evaluation of the position of the shape. The convolutional neural network proposed by Soomro et al. [29] was focused on improving segmentation sensitivity and detecting blood vessels whilst coping with uneven intensities. The proposed deep convolutional neural network (DCNN) [30] was used to segment images of human skin lesions (dermis and epidermal and nontissue regions). The full convolutional network [18] was used to produce coarse results at three different resolutions, which were combined to generate pixel markers. However, in their method, it is difficult to accurately reconstruct a highly nonlinear structure of the boundary of the object. The errors in the segmentation are mainly due to the overlap of tissue boundaries. ResNet [9, 31] has a very deep network architecture, which generates good features in many image classification applications. Lin et al. [32] proposed a RefineNet that incorporates ResNet-101 to enrich visual information.

Unfortunately, existing semantic segmentation methods require a large amount of training data for annotation, which can be difficult to collect in a new medical field. Milletari et al. proposed Hough-CNN [33], which overcame the shortcomings of a small training dataset. The patched multimap method implicitly encoded the anatomical shapes and provides a smooth segmentation profile. However, this method does not capture specific contour information. Ronneberger et al. [5] proposed U-Net, which connects different levels of feature maps, combined with the feature map of the middle layer and thus achieving good performance in biomedical image segmentation. Fabijańska [34] proposed a new semantic segmentation network based on U-Net, which uses the shallower architecture to achieve the same results as the original U-Net. In particular, it requires extensive evaluation of larger image datasets. Based on the U-Net model, Xia and Kulis [35] proposed W-Net that connects two fully convoluted network (FCN) architectures (each one similar to the U-Net architecture) with an automatic encoder. In order to make full use of the advantages of U-Net and discard its disadvantages, a number of stacked U-Net architectures [36, 37] have been proposed. Compared to a single U-Net, stacked U-Net can capture higher-order spatial relationships, and each U-Net can extend the side path for its own prediction, thereby improving prediction accuracy. Tang et al. [38] proposed a coupled CU-Net, through the connections of multiple U-Net of their semantic blocks. CU-Net has the advantages of multilevel top-down and bottom-up reasoning and intermediate supervision; it is parameter efficient and benefits from U-Net's information delivery. However, as the number of convolutional layers increases, it is difficult to achieve convergence if there is no algorithmic guidance during the training.

Although the above methods have made promising progress in segmentation, they cannot be directly used to deal with fluorescent-stained vessels. In our dataset, there are a large number of overlapping vessels with complex structures and multiple shapes. Inspired by the literature, we here propose a new structure, using the dual U-Net model, different from the previous stacked U-Net. Bridging two U-Nets can reduce the training costs and accelerate the convergence of the neural network. Accurate boundary determination can greatly improve the performance of semantic segmentation. However, it is still quite difficult to detect the ISV region with insufficient fluorescence doses. In order to delineate images well, Zheng et al. [39] introduced a CRF-CNN which combined the advantages of both probabilistic and graphical modelling. Conditional random field (CRF) is focused on seeking profile energy intensity information and is applied to output graphs for fine-tuning segmentation. HashishGhosh and Bandyopadhyay [40] put forward image co-segmentation using dual active contours: two contours proceed towards the boundary of the common object until the two contours coincide at the object boundary. Korfiatis et al. [41] proposed a new independent active contour segmentation framework based on two autonomous modules, namely automatic ROI extraction and independent active contours evolution, which segment the ROI image using multiple active contours. Although these contour-based algorithms have made some progress, they are time consuming and difficult to be applied to our network. Hu et al. [42] developed an improved cross-entropy loss function for multiscale CNN for retinal vessel segmentation, and applied CRF to obtain more spatial context information.

## 3. Principles and Methods

**Figure 2:**
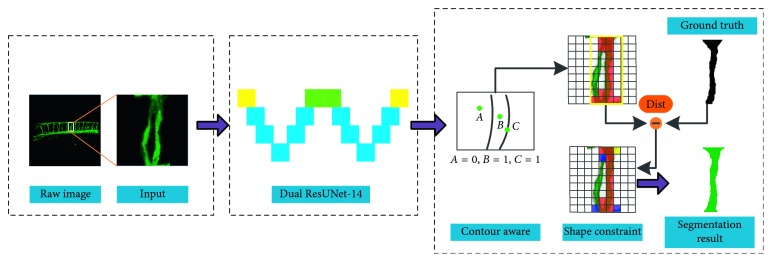
The workflow of the proposed learning methodology.

In this section, we introduce the proposed segmentation method of zebrafish embryonic blood vessels. In view of the difficulties of ISV mentioned above, we have improved the original U-Net model, expanded it into two U-Nets, and combined them together. At the same time, we propose a cross-entropy loss function including contour aware and shape constraints in order to solve the segmentation problem caused by various complex forms and uneven fluorescence intensity. The processing steps are shown in Figure 2.

### 3.1. Framework of the Proposed Approach

**Figure 3:**
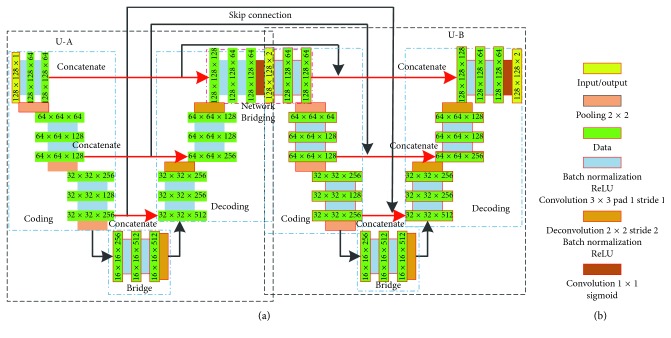
The architecture of the proposed Dual U-Net: (a) network diagram; (b) legends.

The network structure used for foreground segmentation of a zebrafish embryo fluorescent vessel image is shown in Figure 3. We have made some important modifications to the traditional U-Net network. Inspired by Xia and Kulis [35], we first extend the network structure by bridging two U-Nets as our deep neural network architecture; then, we reduce the number of convolution layers in each U-Net. The proposed architecture achieves the same results as the original U-Net and makes the training easier. Finally, we replace the original unit of U-Net with a full preactivated residual unit. Specifically, a large number of feature channels exist in the U-Net structure, which allows the network to propagate the contextual information to higher resolution layers, which is very similar to the residual neural network. He et al. discussed the effects of different combinations in detail in [9] and proposed a full preactivation module to further improve network performance. Combining the advantages of both, we use the full preactivation residual units to replace the original units of U-Net. This residual unit is used to fine tune the weight of the segmentation task. We use concatenation as a bridging method for two U-Nets [43]. In the network, we use an addition for skip connection [44]. If we used concatenation in the skip connection method, it may be beneficial for weight initialization, but would increase the learning complexity of the second U-Net decoder, causing difficulty in convergence.

Similar to the original U-Net architecture, each ResUNet also has two parts: the contraction path (left) and the extension path (right). Our dual ResUNet architecture consists of 14 stacked residual units; each residual unit contains a pair of batch normalization (BN), rectified linear unit (ReLU) and 3 × 3 convolution kernels. To keep the image size constant, we apply a pixel zero padding before each convolution operation starts. After each residual block, we add a 2 × 2 max pooling operation with the step size of 2 pixels for downsampling, doubling the number of feature channels. Correspondingly, in the above sampling process, we use a 2 × 2 convolution kernel with step size 2 to carry out deconvolution, reducing the number of characteristic channels by a factor of two, followed by BN and ReLU, in order to prevent the gradient from disappearing whilst speeding up convergence. The final convolutional layer is followed by the sigmoid layer, which produces two probability graphs. Each pixel is classified as ISV or non-ISV by using a series of convolution, ReLU, and batch normalization layers to determine the characteristics.

### 3.2. Loss Function

For the segmentation task, contour and shape information can effectively reduce the ambiguity of the segmentation region. In this paper, we introduce a novel contour-aware term and a shape-constraint term into the segmentation loss function. Firstly, the contour structure of pixel-to-pixel regions is introduced, which can be considered as a probability contour map. Although the downsampling path can create high-level abstract features, it can lead to the loss of spatial information and abstraction. In the segmentation task, the contour and shape information of the region provides a good complement to the object segmentation. Therefore, contour information and shape constraints were integrated into the U-Net to form a deep segmentation network to accurately segment the ISV in fluorescent images. Segmentation is particularly difficult in the presence of various complex forms and uneven fluorescence intensities. The convolution operation tends to respond to the edge position in the shallow layer of the neural networks. As the network deepens, the detailed structural information is gradually eliminated for convolution and pooling operations. Therefore, it is necessary to consider the linear combination of all the feature maps by the proposed deep networks.

We use the following pixel-wise log loss function to generate prediction results similar to the ground truth by minimizing *W*:

(1) W^=arg minW∑n=1NLXn ∣ yn;W,

where *N* is the number of the training examples, (*X*^(*n*)^ | *y*^(*n*)^) represents the *n*th example in the training set *X* and its ground truth *y*, and *W* is the weight parameters of the network. The resolution of the feature map decreases as *j* increases. Therefore, bilinear interpolation of each feature map is adopted to adjust the original pixel resolution. We define *f*_*j*_(*p*) as the response map of the *j*th pixel *p*, whilst calculating the linear combination *y*(*p*) as follows:

(2) $$\begin{array}{c} y\left(p\right)=\overset{J}{\underset{j=1}{\sum }}w_{j}f_{j}\left(p\right), \end{array}$$

where *p* is the pixel feature, *J* is the number of the network layer, and *w*_*j*_ is the weight of layer *j*. We use weighted cross entropy as our loss function:

(3) $$\begin{array}{c} L=-t\;\log\left(\text{sigmoid}\left(y\right)\right)+\left(1-t\right)\left(1-\text{sigmoid}\left(y\right)\right), \end{array}$$

where *t* is the target labeling and *y* is the predicted value of input. For the segmentation of the ISV image, we can define the weight value by a sigmoid cross-entropy loss for each image as follows:

(4) $$\begin{array}{c} L_{c}\left(X^{\left(n\right)}\;|\;y^{\left(n\right)};W\right)=-\left|\frac{I_{+}}{I}\right|\underset{i\in I_{+}}{\sum }\mu_{i}\left(p^{\left(i\right)}\right)\log\left(y_{i}\left(p^{\left(i\right)}\right);W\right)-\left|\frac{I_{-}}{I}\right|\underset{i\in I_{-}}{\sum }\left(1-\mu_{i}\left(p^{\left(i\right)}\right)\right)\log\left(1-\left(y_{i}\left(p^{\left(i\right)}\right)W\right)\right), \end{array}$$

where

(5) $$\begin{array}{c} \mu \left(p\right)=\left\{\begin{array}{cc} 1, & \text{within contour},\\ 0, & \text{other regions}. \end{array}\right. \end{array}$$

*I*_−_ and *I*_+_ are the subsets of inner and outer contour pixels, respectively, *I*=|*I*_−_|+|*I*_+_|. *μ*(*p*) is the true label.

Further, contour-aware constraint is not available to identify the junction areas of ISV-DA and ISV-DLAV. Therefore, we introduce a shape-constraint term *L*_*s*_ in the loss function and calculate the Euclidean distance between the ground truth and the prediction. We add an additional penalty to the function in order to ensure that the correct segmentation result is obtained.

(6) $$\begin{array}{c} L_{s}\left(X^{\left(n\right)}\;|\;y^{\left(n\right)};W\right)=\text{Dist}\left(\hat{Y},Y_{\text{GT}}\right). \end{array}$$

**Figure 4:**
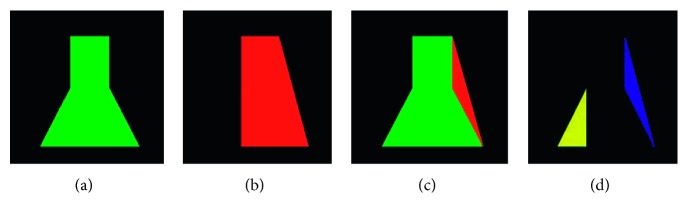
Contour-aware and shape-refinement schematic diagram: (a) ground truth; (b) predicted result; (c) compared with ground truth *Y*_GT_ (green) and predicted result $\hat{Y}$ (red); (d) false positive (blue) and false negative (yellow).

Specifically,

(7) $$\begin{array}{c} \text{Dist}\left(\hat{Y},Y_{\text{GT}}\right)=-\overset{N}{\underset{i=1}{\sum }}q_{i}\;\log\left(u_{i}\right), \end{array}$$

where Dist(·) denotes the Euclidean distance between the predicted result $\hat{Y}$ and the ground truth *Y*_GT_, when the *i*th result is the correct class *q*_*i*_=1, otherwise *q*_*i*_=0. *u*_*i*_ is the *i*th element of the class scores.  Figure 4 shows the comparison results.

We show the ground truth (Figure 4(a)) and the predicted result (Figure 4(b)) via the network. By calculating the Euclidean distance between the ground truth (green) and the prediction (red) (Figure 4(c)), we add the additional penalty to the function. In the first few training epochs, because the prediction results differ greatly from the ground truth, the value of Dist is also large. As the training progresses, Dist decreases. Therefore, the proposed shape constraint implicitly forces the network to learn to predict the masks faster.

Subsequently, the shape-refinement terms we propose are as follows:

(8) $$\begin{array}{c} \hat{W}=\underset{W}{\arg\text{ min}}\overset{N}{\underset{n=1}{\sum }}\left(L_{c}\left(X^{\left(n\right)}\;|\;y^{\left(n\right)};W\right)+L_{s}\left(X^{\left(n\right)}\;|\;y^{\left(n\right)};W\right)\right). \end{array}$$

Our goal is to evaluate the parameter *W* with a minimal overall loss in order to produce an accurate foreground ISV region. The network weight *W* is randomly initialized from the Gaussian distribution. We regularize the weight decay of 5 × 10^−4^ in order to punish *W* during the backpropagation.

## 4. Experiment Results and Metrics

In this section, we conduct extensive experiments to assess the performance of the proposed methods in zebrafish ISV classification and explain the details of the experiments. Both quantitative and qualitative results are discussed.

### 4.1. Experimental Setup and Datasets

**Figure 5:**
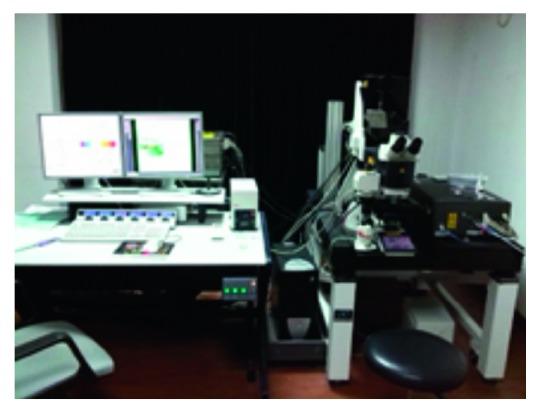
Leica TCS SP5 laser confocal fluorescence microscopy system.

All the zebrafish vascular images are provided by the Key Laboratory of Neurogenesis, Nantong University. A series of confocal stack of 25 images of the trunk region is obtained in a time interval of 30 minutes. Each slice is of 2252 × 1265 pixels and 24-bit grey-level matrices. Confocal imaging is performed with a Leica TCS-SP5 LSM. The instruments are shown in Figure 5. Zebrafish are bred and raised according to the standard procedures on a 14-hr light/10-hr dark cycle at 28 degrees Celsius. Zebrafish with 35–72 hours postfertilization (hpf) life are suitable for observation, whose body length is 2–3.5 mm.

There are 672 2D images, containing 6842 ISV patches. In order to reduce the overfitting of the classification architecture and to improve the generalization ability of the classification tasks, we carry out data augmentation of the image blocks, mainly using the affine transform combination operation (mirror and flip), expanding the dataset by four and generating a certain number of subimages (27368 training patches). At the same time, these 672 images are divided into three groups for training, testing, and verifying. Manually labeling the zebrafish foreground ISV is extremely time-consuming and labor-intensive. We invited four research assistants with more than 3 years of research experience to label our dataset. We also invited a senior expert with 10 years of research experience for strict quality control on the results of manual labeling. All annotation is subject to verification by the senior expert.

### 4.2. Training

Our model is implemented based on the open source deep learning library of PyTorch [45] using Python 3.6. During the training, we perform five cross-validation sessions, trained with 60% of the images, tested with 20% of the images, and verified with the remaining 20%. We conducted a series of comparative experiments to obtain the best input size. A low-resolution patch can shorten the training time, but it also causes a big problem and cannot provide more detailed information. The proposed Dual ResUNet-14 is trained using 128 ∗ 128 pixel patches. This comparison provides the best accuracy for the CNN pixel classification in the experiments. The patches are randomly extracted from the training images. The experiment runs on a computer with a 3.2 GHz Intel Xeon CPU and 64 GB RAM. Meanwhile, we use two GPUs (Nvidia Geoforce GTX1080Ti) to accelerate part of the model training process. The learning rate is originally defined as 0.001, and it was reduced by half when the relative improvement of *L* was less than 1% of the average loss of the first 10 epochs. When the relative difference is less than 10^−4^ or reaches a maximum of 150 epochs, the optimization stops.

### 4.3. Segmentation Overview

**Figure 6:**
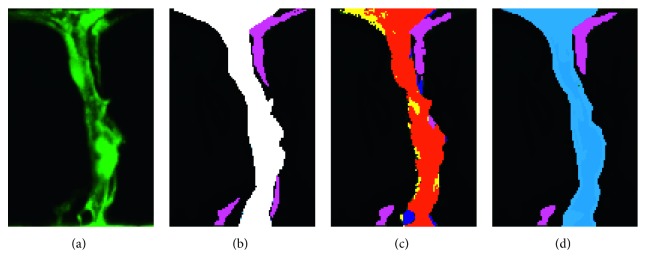
Dual U-Net output schematic diagram: (a, b) the input image and ground truth: foreground ISVs (white) and background ISVs (pink); (c) the output of the network: true positive (red), false positive (blue), and false negative (yellow); (d) the final result of segmentation.

Due to unclear occlusion and photography, the shape of background blood vessels cannot be extracted well. We choose only the foreground blood vessels as the basis for segmentation. In actual observation, abnormal blood vessels appear as irregularities such as bifurcation, adhesion, and shortness, which pose great challenges to our segmentation. Furthermore, uneven distribution of fluorescence intensity in blood vessels also reduces the segmentation accuracy of the network. In order to evaluate the performance of our proposed network, we conduct a comprehensive and thorough comparison.  Figure 6 is our proposed schematic diagram.

### 4.4. Network Structure

**Figure 7:**
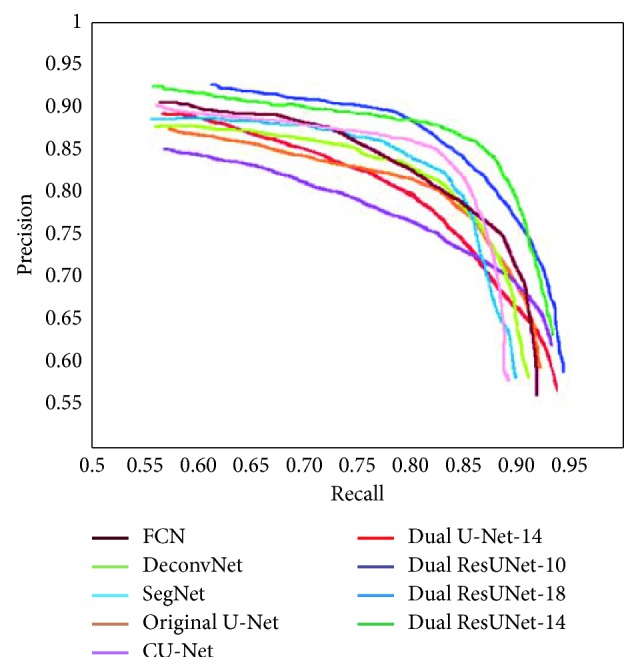
Comparison of PR-curves of our improved methods.

Dual ResUNet is an end-to-end network for the segmentation of zebrafish fluorescent vascular models. Our network combines the advantages of U-Net and ResNet to replace the original U-Net unit with a fully preactivated residual unit to form a new network structure. In theory, the deeper the network, the stronger the representation ability, and the more training data that can be processed, but the training algorithm may not support it. By changing the number of network units, we obtain three full preactivated Dual ResUNets (unit numbers 18, 14, and 10, respectively) and a Dual U-Net (14 U-Net origin units). In order to reach a balance between network accuracy and training complexity, we conduct extensive comparative experiments to obtain the best-performing network structure, including several solutions proposed by us, and some classic segmentation network models. It should be noted that the comparison of these methods is carried out and our proposed loss function is with the contour-aware term and shape-constraint term.  Figure 7 shows the PR curves of the improved methods on our datasets. From Figure 7, we can see that the results reflect the fact that the proposed Dual ResUNet-14 has higher accuracy to some extent. The extensive qualitative and quantitative comparisons are made to show the performance.

**Figure 8:**
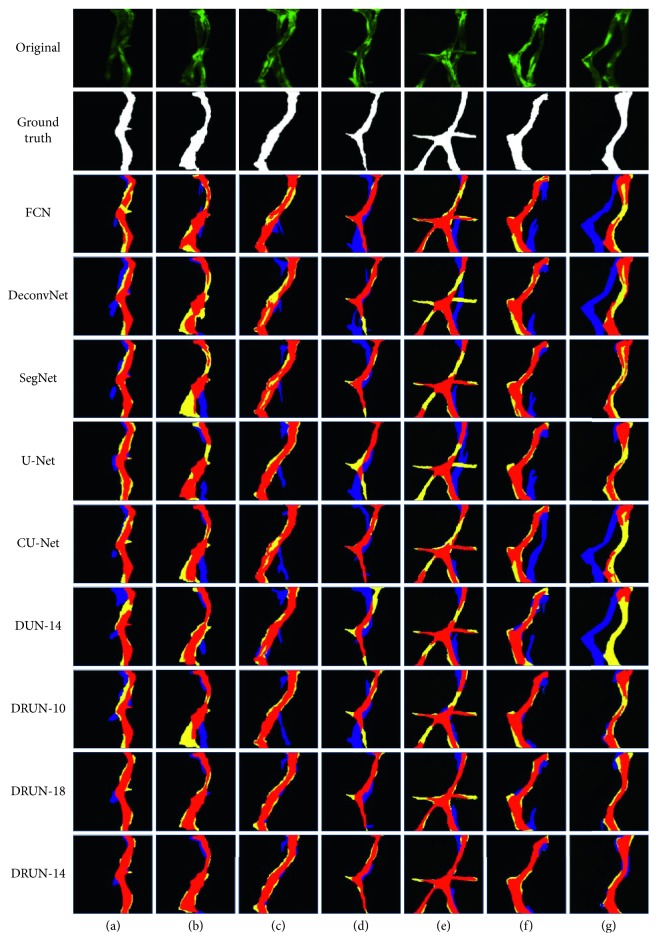
Segmentation results: true positive (red), false positive (blue), and false negative (yellow). DUN: Dual U-Net; DRUN: Dual ResUNet.

An example of qualitative assessments is shown in Figure 8. We choose a typical set of blood vessel models. Figure 8(a) shows some examples of segmentation in which almost all the methods correctly segment the FV, but DUN-14 (Dual UNet-14) does not capture contour information well in the case of low IRF doses in the ISV. Figures 8(b) and 8(c) show examples where the fluorescence intensity of the BV is higher than that of the partial FV, resulting in many network models giving incorrect results. Figure 8(d) shows the similar fluorescence dose on FV and BV. Most of the frames have different degrees of segmentation errors. Fortunately, both DRUN-18 (Dual ResUNet-18) and DRUN-14 can correctly identify the FV, and the effect of DRUN-14 is more significant. Figure 8(e) shows a situation where a FV branches, but the fluorescence dose is low and many network models do not capture this detail. Unlike the others, our DRUN-14 still performed well in the areas of the ISV with similar fluorescence doses. Figures 8(f) and 8(g) show the situation in which there is no overlap between the two ISVs. Compared with other frameworks, DRUN-14 can effectively detect the areas with low fluorescence dose and correctly segment them. Overall, our proposed Dual ResUNet-14 produces better segmentation results than its counterparts. Qualitatively, we believe that Dual ResUNet-14 produces the best segmentation results.

During the test phase, it takes about 0.5 seconds to generate the predicted results. Figure 8 shows that both U-Net and its improved segmentation method are capable of detecting the foreground ISVs. The next step is to make a quantitative assessment. These predictive masks are compared with the ground truth, and the relevant measurement metrics are shown in Table 1.

Table 1 reports the average median (med.), mean, and standard deviation (Std) of the results over the 672 zebrafish vessel-model datasets. Of the classic segmentation models, SegNet performs best. Interestingly, the simplest version of FCN performs well. The improved segmentation network based on U-Net is superior to the classical segmentation network. In the improved network with the best performance, it achieved a 5.9% increase in terms of the mean accuracy, and the Dice similarity coefficient achieved 0.036 improvement compared to the classic segmentation model. At the same time, the standard deviation of both accuracy and Dice is low, which indicates that our residual segmentation network is relatively stable and can greatly correct the vascular segmentation errors. The difference is small quantitatively but these improvements are statistically significant, tested at a 5% significance level according to a paired *t*-test. In other words, the network segmentation performance is statistically improved.

**Figure 9:**
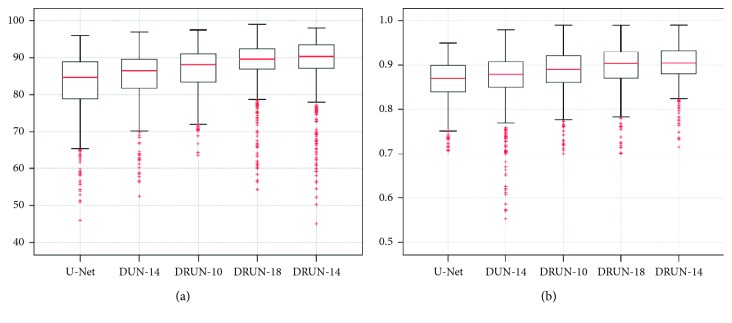
Boxplots of the quantitative metrics: (a) accuracy (%); (b) Dice. DUN: Dual U-Net; DRUN: Dual ResUNet.

We compare our proposed network architecture against the others. The box diagram for quantitative measurements is shown in Figure 9. It can be seen that the mean and median of pixel-level accuracy and Dice are higher than those of the other schemes, indicating that the regularization ability of the new contour-aware term is positively contributing to the segmentation. The shape-constraint term that we introduced is addressing the overlapping regions where the ISV contours are difficult to segment. We conclude that this novel term provides comparable results in almost all the cases.

We also investigate the impact of different patch sizes on our network. Since the U-Net structure has no specific requirements on the input image size, it is usually used to process the entire image. We considered the original fluorescent blood vessel image as the input data, but the predictions obtained are significantly poorer (Table 2), which could not be improved even with more extensive training. We applied opencv3.1 + Visual studio 2015 to adaptively crop the labeled images to ensure that each image contains a pair of ISVs with a clipping height ranging from 120 to 160 pixels. Each image is converted into square images by padding an appropriate number of zeros to the images. Applying our network to specific images can better capture local features. The impact of the patch size on the network performance is summarised in Table 2.

We can see that, during the training process, as the size of the input image increases, the accuracy of Dual ResUnet-14 on the training and verification sets increases. However, on the test dataset, the highest accuracy is obtained for the images of 128 × 128 pixels. Compared to larger sizes, the training results are only slightly worse and take less time. Therefore, in the proposed method, the image size of 128 × 128 pixels is used.

### 4.5. Comparison of Different Loss Functions

**Figure 10:**
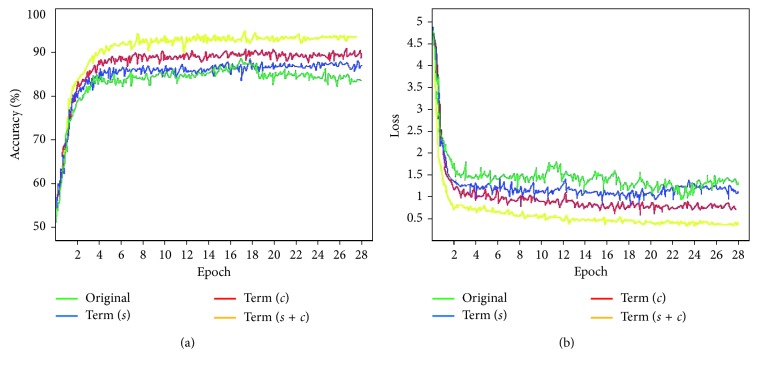
Comparison of loss functions with different constraints: (a) model accuracy; (b) model loss. Original: weighted cross entropy; Term (*s*): adding shape-constraint term; Term (*c*): adding contour-aware term; Term (*s* + *c*): adding shape-constraint term and contour-aware term.

In order to validate the proposed loss function, we set up four comparison experiments: (1) weighted cross entropy without any improvement; (2) introduction of a contour-aware term; (3) introduction of a shape constraint; (4) introduction of contour-aware and shape-constraint. This is shown in Figure 10. As can be seen from Figure 10, a loss function with the term (*s* + *c*) (i.e., contour aware and shape constraint) provides significant improvement. We use validation sets to monitor and adjust the hyperparameters during the training. The proposed network achieves convergence after 7 epochs. After adjusting the model and optimizing all the hyperparameters, we train the final model of all the data using five-fold cross validation. After the introduction of shape constraint, our network does not achieve further improvement. However, significant differences can be observed in the correctly segmented images. In order to fully demonstrate the validity of the introduction of shape-constraint terms in our network, we choose 1000 images with more complex ISV shapes.  Table 3 shows the average metric for adding shape constraints under complex conditions.

**Figure 11:**
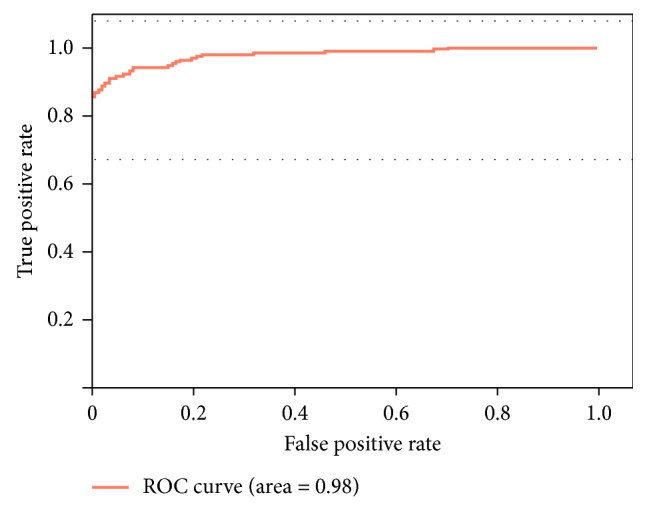
Receiver operating characteristic (ROC) of the segmentation.

We only compared pixel accuracy and Dice, achieving 2.1% and 0.11 improvements over 0.11% and 0.07 of the entire dataset, respectively.  Figure 11 shows several examples of these images.

**Figure 12:**
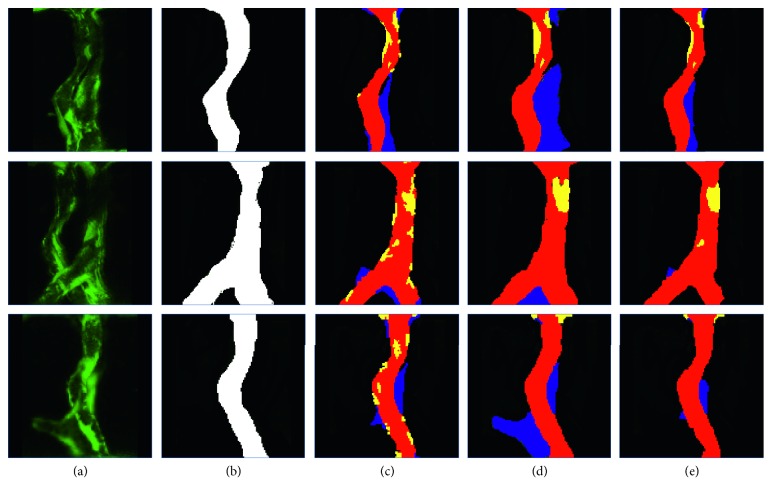
The plot of pixel-level accuracy and loss over the epochs for the Dual ResUnet-14 model trained on the dataset: (a) original; (b) ground truth; (c) DRUN-14 (*s*); (d) DRUN-14 (*c*); (e) DRUN-14 (*s* + *c*). DUN: Dual U-Net, DRUN: Dual ResUNet.

From Figure 12, we can see that our shape constraint plays a significant role in the overlapping areas of the ISV.  Figure 11 shows the ROC curve of zebrafish vascular image segmentation using the Dual ResUnet-14 model. The relationship curve indicates that the result is indeed accurate. It is worth noting that the model achieves 88.91% accuracy and 0.98 AUC, which indicates that the proposed architecture is a promising method for segmenting new data.

**Figure 13:**
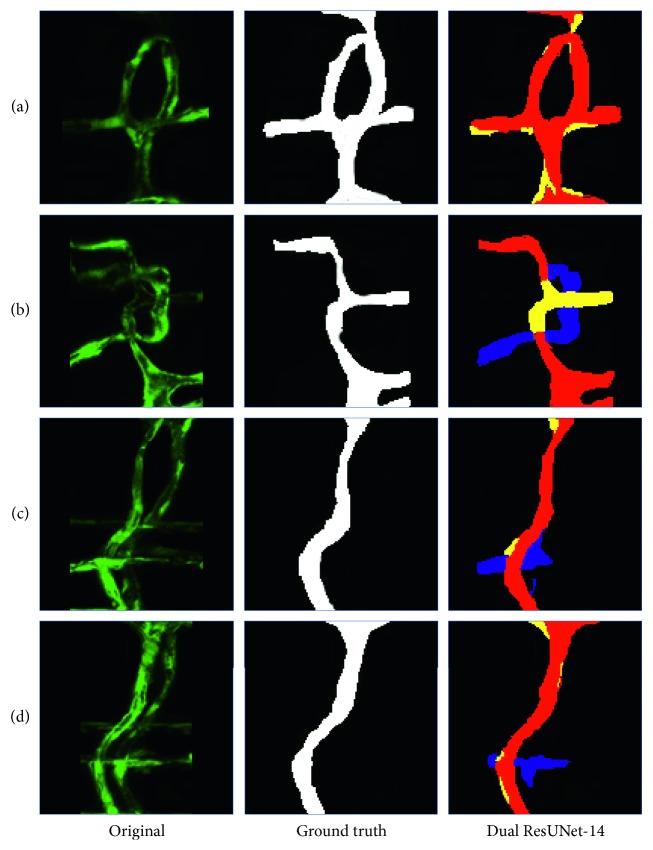
Segmentation results: true positive (red), false positive (blue), and false negative (yellow).

As can be seen from Figure 8, Dual ResUNet-14 with the contour-aware and shape-constraint term performs well compared to the other network architectures, making the foreground ISV segmentation more accurate. However, there are still many examples of poor segmentation due to the presence of zebrafish embryo spinal cord collateral vessels and serious adhesion caused by ISV overgrowth, shown in Figure 13. Most of the abnormal values are caused by low fluorescence intensity in the image. Figure 13(a) is the case of bifurcation, and despite the presence of the spinal cord collateral vessels, our segmentation network can still obtain accurate results. Figure 13(b) is the case of adhesion, and the intricacies of the blood vessels cause large errors in the results. In Figures 13(c) and 13(d), there is a deviation in the network output due to the presence of the spinal cord collateral vessels.

## 5. Conclusion

Automated segmentation of zebrafish vessels has greatly improved the efficiency of clinical work. In this paper, a new automatic segmentation framework for zebrafish fluorescence vessel images was proposed by using a new deep learning technology. It is known that it is difficult to detect and segment overlapping regions caused by uneven distribution of the fluorescence intensity. To solve these issues, we have proposed a novel ResUNet-14 ISV segmentation architecture which combines the full preactivation residual network units and U-Net architecture and introduced a shape-constraint term in the contour-aware loss. By comparing the classical segmentation network with some improved U-Net-based networks, the accuracy of the proposed DRUN-14 is higher than that of other segmentation network models, reaching 88.91%. Our automatic zebrafish embryo fluorescence vessel image segmentation framework can generate the final Dice similarity coefficient of 89.2%. However, our framework does not perform well in bending and sticking regions, which is the problem we need to address in future work.

## Figures and Tables

**Table 1:** Quantitative metrics for ISVs segmentation.

	Accuracy (%) (TP+TN/TP+TN+FP+FN)				Dice (2TP/2TP+FN+FP)			
	Med.	Mean	Std.	*P* value	Med.	Mean	Std.	*P* value
FCN	85.23	83.21	10.31		0.870	0.843	0.131	
DeconvNet	83.62	82.77	10.20		0.852	0.839	0.140	
SegNet	85.61	84.62	8.55		0.858	0.841	0.132	
U-Net	84.67	83.01	8.62		0.871	0.856	0.123	
CU-Net	88.30	85.45	6.22		0.885	0.879	0.092	
Dual UNet-14	86.51	85.14	7.34		0.880	0.870	0.091	
Dual ResUNet-10	88.11	86.48	5.47		0.891	0.875	0.068	
Dual ResUNet-18	89.61	88.80	3.94		0.901	0.885	0.050	
Dual ResUNet-14	90.34	88.91	3.21	*<*10^*−12*^	0.904	0.892	0.044	*<*10^*−12*^

**Table 2:** The influence of patch size on accuracy.

Patch size	Training	Validity	Testing
Original	0.684	0.681	0.683
32 × 32	0.812	0.823	0.809
64 × 64	0.852	0.858	0.846
128 × 128	0.889	0.887	0.90
160 × 160	0.901	0.90	0.895

**Table 3:** Comparison of different loss functions under complex conditions.

Average quantitative metrics		
	Pixel-wise accuracy (%)	Dice coefficient
Original	78.54	0.803
Term (*s*)	80.44	0.815
Term (*c*)	87.67	0.869
Term (*s* + *c*)	88.91	0.892

## Data Availability

The zebrafish data used to support the findings of this study were supplied by the Co-innovation Center of Neuroregeneration, Jiangsu Key Laboratory of Neuroregeneration under license and so cannot be made freely available. Requests for access to these data should be made to Kun Zhang at zhangkun_nt@163.com.
